# Perceptions of Nurses at a University Hospital regarding Personal Values and Leadership

**DOI:** 10.1590/0034-7167-2025-0102

**Published:** 2026-10-26

**Authors:** Chennyfer Dobbins Abi Rached, Giovanna Micucci Pires Amaral, Maria Luiza Prado Uelze, Bruna Franco Massa

**Affiliations:** I Universidade de São Paulo. São Paulo, São Paulo, Brazil

**Keywords:** Nursing, Nurses, Leadership, Social Values, Value-Based Health Care., Enfermería, Enfermeras y Enfermeros, Liderazgo, Valores Sociales, Atención Médica Basada en Valor.

## Abstract

**Objectives:**

to understand nurse managers’ perceptions regarding personal values and leadership style.

**Methods:**

qualitative field research. Nineteen nurses holding management positions at a university hospital in São Paulo participated. Five focus groups were conducted with trigger questions addressing leadership styles, personal values, and the institutional context. Data were analyzed using the Discourse of the Collective Subject method.

**Results:**

the prevalence of the relational style, the influence of personal values on leadership style, and conflicts between personal values and working conditions were evident.

**Final Considerations:**

personal values are reflected in leadership, with the relational style being associated with team engagement and humanization in care.

## RESUMEN

**Objetivos:**

comprender las percepciones de las enfermeras gestoras respecto a sus valores personales y estilo de liderazgo.

**Métodos:**

investigación de campo cualitativa. Participaron diecinueve enfermeras con cargos directivos en un hospital universitario de São Paulo. Se realizaron cinco grupos focales con preguntas clave que abordaron estilos de liderazgo, valores personales y el contexto institucional. Los datos se analizaron mediante el método del Discurso del Sujeto Colectivo.

**Resultados:**

se evidenció la prevalencia del estilo relacional, la influencia de los valores personales en el estilo de liderazgo y los conflictos entre estos y las condiciones laborales.

**Consideraciones Finales:**

los valores personales se reflejan en el liderazgo, y el estilo relacional se asocia con el compromiso de equipo y la humanización de la atención.

## INTRODUCTION

The globally disseminated concept of leadership is the ability to influence others in pursuit of common goals. John Maxwell, one of the most influential authors on leadership, states that “The true measure of leadership is influence-nothing more, nothing less”^(1)^. Various theories and models of leadership define the concept in the same way^(2-5)^. Authors and researchers in the field of leadership and management argue that leaders’ personal values shape the organizational culture and influence leadership style^(6-9)^.

The set of personal beliefs and values of leaders explains their decisions and behaviors; in other words, it functions as an internal guide. Among the ways in which personal beliefs and values influence leaders, decision-making stands out, and a leader who values integrity prioritizes ethical decisions. During crisis situations, a leader’s values determine the course of action. Leaders who value transparency communicate openly, while those who prioritize security adopt cautious stances^(9,10)^.

Leaders who value self-direction and benevolence adopt a more participative and democratic leadership style, building strong and supportive relationships with their teams and fostering a positive and collaborative work environment. In contrast, those who value power tend to adopt a more authoritarian and directive style^(6-8)^.

Personal values offer insights into leaders’ behavior and, in turn, their impact on professional practice^(10,11)^. In the context of nursing, leaders who value empathy are more likely to practice active listening, which is essential in interacting with patients, families, and healthcare teams^(9,12)^.

Reflecting on the effect of personal values on leadership style allows for the recognition of biases or personal preferences that can generate conflicts or tensions. Values guide interactions, which favors dialogue and collaborative work, contributing to greater employee engagement, with positive effects on care^(10,13)^.

Although the phenomenon of leadership has been extensively studied in the field of organizational behavior, there is a gap regarding the relationship between leadership and personal and organizational values. Therefore, the focus of this study is to understand nurse managers’ perceptions in a public university hospital regarding personal values and leadership styles. The investigation is based on Schwartz’s Theory of Basic Human Values and Values-Based Leadership, with the aim of contributing to the strengthening of conscious, ethical management practices aligned with nursing professional identity. Thus, a literature review is presented that aims to conceptualize the structure of personal values and their influence on individual behavior, as well as the development of Values-Based Leadership.

### Schwartz’s Theory of Basic Human Values

The Theory of Basic Human Values establishes values as “(a) concepts or beliefs, (b) about desirable end states or behaviors, (c) that transcend specific situations, (d) guide selection or evaluation of behavior and events, and (e) are ordered by relative importance”^(14)^.

According to Schwartz (1992), values are classified into ten motivational types: 1) universalism; 2) benevolence; 3) conformity; 4) tradition; 5) security; 6) power; 7) achievement; 8) hedonism; 9) stimulation; and 10) self-direction. These values exhibit varying degrees of overlap, which consequently influences how the individual behaves and reacts to the presented contexts^(8,15)^.

Therefore, an individual’s behavior is influenced by their set of personal values, and thus, a leader’s framework of beliefs and values impacts their conduct^(9)^. Therefore, leaders who are attentive to the values of their team members contribute to greater satisfaction and engagement. Thus, institutional values must be in harmony with the values of both the leader and the employees^(7,16,17)^.

### Values-Based Leadership

Thinkers such as Aristotle and Kant discussed the importance of ethical principles and virtue, influencing the notion of moral leadership, which consists of the idea that leaders should act in accordance with ethical and moral values^(7)^.

Values-Based Leadership emerged as a significant approach in management and leadership studies in the 1990s, driven by a desire for authenticity and ethics in the workplace. It focuses on the importance of leaders being guided by core values, influencing thinking about how leaders can be effective and inspiring when they possess positive values^(4,7)^.

In 1978, James MacGregor Burns published his seminal work “Leadership”, which introduced the distinction between transformational and transactional leadership. Transformational leadership, which is based on values and inspires followers to achieve high goals, was one of the first forms of leadership to emphasize the importance of personal values^(2)^.

Bernard Bass expanded and refined Burns’ ideas, especially in the context of transformational leadership. The emphasis on vision and values as factors that motivate and inspire followers to achieve high performance solidified the theoretical foundation of Values-Based Leadership. Leaders in the healthcare sector began adopting this model through a shared vision and core values in support of patient-centered care^(2)^.

In the 2000s, authentic leadership gained prominence, with researchers like Bill George, author of “Authentic Leadership” (2003), arguing that effective leaders are those who act with authenticity and consistency in relation to their personal values and principles. Authentic leaders are transparent and build relationships with empathy, respect, and mutual trust^(13,17)^.

Servant leadership, whose author is Robert K. Greenleaf in the essay “The Servant as Leader” (1970), influenced Values-Based Leadership by proposing that effective leadership embodies values such as empathy and respect, demonstrating commitment to the well-being of its followers^(3)^.

Therefore, Values-Based Leadership emerges as a response to the demands for greater ethics, authenticity, and effectiveness in leaders. Ethical and moral principles, as leadership practices, reinforce the importance of leaders being guided by core values that shape their actions. This approach has been influenced by theories of transformational, authentic, and servant leadership, as well as by cultural changes and organizational contexts, with emphasis on integrity and responsibility^(16,17)^.

Furthermore, changes in social and cultural expectations regarding corporate social responsibility and sustainability have also contributed to the popularization of Values-Based Leadership, as organizations have become more aware of their socio-environmental impact^(4,7,10)^. The Values-Based Leadership Theory highlights that effective leaders act in accordance with values and principles, fostering trust and engagement among followers^(7,16,17)^.

In the healthcare field, Values-Based Leadership has emerged as a response to the search for best management practices and for ethical and humanized care. The complexity and direct impact of leadership decisions on people’s health and well-being have made it essential to adopt an approach that prioritizes strong values and ethical principles^(2,9)^.

Faced with cases of malpractice, negligence, and lack of transparency, the desire of leaders for values such as safety and quality of care has become more evident. Values-Based Leadership has been applied with a focus on patient-centered care. Such models emphasize the importance of treating patients with dignity and respect, promoting the active participation of patients in their own health decisions. In this regard, nurses’ values influence the care received by patients and their families^(2,9)^.

## OBJECTIVES

To understand nurse managers’ perceptions regarding personal values and leadership styles.

## METHODS

### Ethical aspects

This study was approved by the Research Ethics Committee of the School of Nursing at the *Universidade de São Paulo* (Certificate of Presentation for Ethical Consideration (CAAE) 51073421.2.1001.5392) and the University Hospital (CAAE 51073421.2.3001.0076). All participants signed the Informed Consent Form, as recommended by Resolution 466/12 of the Brazilian National Health Council.

### Study design

This is an exploratory qualitative study, based on the COnsolidated criteria for REporting Qualitative research, focusing on understanding nurse managers’ perceptions of personal values and leadership style. Data collection and analysis followed the Discourse of the Collective Subject (DCS) method^(18)^, allowing the representation of central collective ideas through individual speeches.

### Methodological procedures

Participants were invited using purposive sampling criteria via email linked to the continuing education service. Two nurses declined the invitation, resulting in 19 nurses holding formal leadership positions (coordinator, head, or director of a nursing department) at a university hospital located in São Paulo, Brazil, with a capacity of 258 beds. The inclusion criterion was holding a leadership position in nursing care, with a minimum of one year of experience in the role. It is worth noting that there were no dropouts during the research and that participants had no prior relationship with the research team.

### Instruments and data collection

Data collection took place in two stages. Initially, two instruments were applied: (1) the Management Style Assessment Scale (MSAS)^(19)^, composed of 19 items on a Likert scale (1 to 5), which identifies the predominant leadership styles (relationship, task or situation); and (2) the Culture and Value Analysis Tool (C-VAT)^(20)^, an ipsative scale based on Schwartz’s Theory of Basic Human Values, which classifies values into four dimensions (work, relationships, control, and thinking), with sub-dimensions and scores from 5 to 20 points.

Subsequently, five focus groups were conducted, each lasting an average of 90 minutes, on a weekly basis in a reserved space at the institution. The sessions were moderated by experienced researchers with teaching experience and non-participant observers, all from the nursing field. It is noteworthy that the research team received training from the research coordinator. During the groups, field notes were taken and audio recordings were made. A verbatim transcription of the focus group content was then performed.

The guiding questions were “Which leadership styles do you identify as most frequent in nursing?”, “Which personal values influence your leadership style?”, “Do you perceive a relationship between your personal values and the leadership style adopted?”, “How do these values impact your relationship with the team?”, and “How does the institutional environment favor or limit the expression of these values?”. The meetings also included moments of reflection based on the individual results of the applied scales. There were no repetitions of the interviews, and data saturation was discussed among the research team members.

### Data analysis

The transcripts of the focus groups were analyzed using the DCS method, as proposed by Lefèvre and Lefèvre^(18)^, which allows the identification of key expressions, central ideas and the elaboration of synthesis discourses representative of the collective. This approach was triangulated with the results obtained from the MSAS and C-VAT scales, which allowed for a more in-depth interpretation of the perceptions and values expressed by participants. Data triangulation was employed to ensure greater rigor and interpretative validity. It should be noted that the themes that emerged throughout the analysis were derived from the data.

### Methodological rigor

To ensure analysis reliability, credibility, transferability, dependence, and confirmation criteria were used, according to the Lincoln and Guba^(21)^ framework. All stages were recorded in a field notebook, with double data coding and independent review of transcripts. The transcripts were not given to participants, but the feedback of the speeches to participants, for member checking, was carried out at the end of the meetings, allowing reflections based on the findings and also feedback from the participants to the research team.

## RESULTS

Analysis of the focus groups revealed three main axes related to nurses’ perception of leadership styles and personal values: (1) prevalence of the relational style; (2) influence of personal values on leadership practice; and (3) tensions between personal values and institutional context.

Regarding the first axis, most participants pointed to the relationship-based leadership style as the most evident and desirable in nursing practice, as it promotes a welcoming, empathetic, and collaborative work environment. The statements highlighted empathy, listening, respect for differences, and care for others as the foundations of this leadership. “*Cooperation and respect transform teams, motivating collaboration among everyone*”, reported Subject 12, demonstrating an understanding of the relational value as structuring care.

In contrast, a smaller group valued the task-focused management style, related to the need for control, efficiency, and standardization of processes. Participants like Subject 5 argued that this model ensures compliance with deadlines and protocols, which directly impacts patient safety. Although less frequent, this style was understood as complementary to relational leadership, especially in high-demand contexts.

Situational style was also recognized, especially among managers with high flexibility scores on the C-VAT scale. The discourses emphasized the need to adapt a leader’s behavior to the team’s reality and care context, evidenced by statements such as that of Subject 17: “*The leader needs to be flexible and make decisions according to the moment, the situation, and the profile of the team*”.

The second analytical axis demonstrated the strong influence of personal values on leadership practice. The “work” dimension obtained the highest scores in the C-VAT, with emphasis on the “quality” sub-dimension, highlighted as an ethical and technical pillar of leadership in nursing. The “quality” value was associated with both patient care and the organization of work routines.

The “relationships” dimension was also highly valued, especially the “sociability” sub-dimension. Participants highlighted that effective communication, empathy, and acceptance promote a positive organizational climate, fostering team engagement. “*Sociability facilitates the exchange of knowledge and best practices with the multidisciplinary team*”, stated Subject 21.

In contrast, the “status” sub-dimension obtained the lowest scores, highlighting the perception of devaluation of the profession. Statements such as that of Subject 3 (“*Society does not value nurses, and we do not value ourselves*”) indicate the internalization of this professional stigma, which can negatively impact nurses’ identity and self-image.

Finally, the third axis indicated a tension between nurse leaders’ personal values and the institutional conditions that limit their expression. Although participants recognize the importance of listening, planning, and providing support, they mentioned that work overload, lack of autonomy, and a hierarchical organizational culture hinder the full experience of these values in daily life.

These results point to the need for institutional programs that promote alignment between professionals’ values and organizational culture, creating spaces for the exercise of leadership consistent with the principles of nursing and humanization of care.

## DISCUSSION

The findings of this study indicate that relationship-focused leadership is predominant among nurse managers at the university hospital investigated. Participants’ statements highlighted that values such as empathy, affection, respect, and trust shape how these professionals lead their teams. These perceptions align with the literature on transformational leadership, which emphasizes empowerment, autonomy, and recognition of individual contributions as central elements of effective management^(2,22)^. For instance, Subject 20’s speech, which highlights the importance of affection and respect, directly reflects the essence of this approach.

Although relational style was largely advocated, some managers pointed to task-focused leadership as the most appropriate in contexts that demand strict adherence to deadlines and goals. Subject 1’s and Subject 5’s statements illustrate this perspective, emphasizing the relevance of standardization and safety in care. This profile is close to transactional leadership, characterized by results orientation, use of clear instructions, and focus on performance^(23,24)^.

Situational leadership was also mentioned among participants, especially among those who demonstrated high levels of flexibility on the C-VAT scale. Subject 8’s and Subject 17’s speeches evidenced this adaptability, reaffirming Hersey and Blanchard’s theory^(13)^, according to which the leader’s effectiveness lies in the ability to adjust the style to the team’s needs and environment conditions.

Analyzing the results of the C-VAT scale, the “work” and “relationships” dimensions stood out. The value of “quality”, strongly cited in the statements, appears as a guiding principle for nurse leaders’ practices, and is also a professional ethical duty according to the Nursing Code of Ethics^(25)^. Subject 9, for instance, directly associated quality with good patient relationships and treatment effectiveness.

In the “relationships” dimension, the “sociability” sub-dimension was highly valued. Participants’ statements reinforced that sociability fosters communication, acceptance, and the exchange of experiences in a multidisciplinary environment. This aligns with studies that point to empathy as a key element in nursing leadership, communication with patients and their families, and the humanization of care^(12,26-28)^. Considering that a leader influences how relationships are established, it is worth emphasizing that such relational aspects also impact patient safety^(29,30)^.

On the other hand, the “status” sub-dimension received the lowest scores, reflecting a perception of low social value placed on the profession. Subject 3’s and Subject 15’s statements expressed a feeling of invisibility and a struggle for recognition, supporting studies that denounce the stereotypes and devaluation of nursing in the media and in society^(31-33)^.

The “planning” sub-dimension, in turn, was well evaluated, being considered essential for the organization of care and for patient safety. The data suggest that the nurse leader values planning as a comprehensive part of the Nursing Process, an aspect also supported by the regulations of the Federal Nursing Council^(34)^. In this regard, nurse leaders influence not only the care provided, but also how it is received by patients and their families, considering that safer and more well-founded care results in greater recognition of the nursing team^(35)^.

### Study limitations

This study has limitations inherent to its qualitative design and the context investigated. The sample was purposive and comprised 19 nurses in managerial roles from a single university hospital, which limits the transferability of the findings to other services, regions, and governance models. Data collection through focus groups may have been influenced by group dynamics, perceived hierarchy, and social desirability, potentially encouraging consensus and inhibiting divergent viewpoints, despite strategies adopted to ensure rigor. This is a cross-sectional snapshot that captures perceptions at a specific point in time and does not allow inferences about changes over time or causal relationships between personal values and leadership styles.

### Contributions to nursing, health, or public policy

Reflecting on personal values and how they influence nurse leaders’ behavior allows for analysis of personal experiences and thoughts, as well as enabling a rethinking of personal and professional values.

The meetings triggered a process of self-awareness among the nurses regarding their values, since rethinking them is a reflective process that takes time. Values impact the leadership style adopted and, consequently, the organizational culture and climate. Knowing one’s own values and having the opportunity to rethink them fosters transformation.

For an organization, understanding the personal values profile and leadership style of its employees can represent a starting point for reflecting on the direction to be followed, contributing to the planning of organizational culture and climate management programs, as well as to the development of Values-Based Leadership focused on identified results.

## FINAL CONSIDERATIONS

The findings of this study show that personal values influence the leadership style adopted. The most prominent values-empathy and sociability-are directly associated with the relational leadership style, which proved to be predominant among nurses. These values demonstrate the potential to strengthen the organizational climate and improve the quality of care provided, especially with regard to the humanization of care.

In addition to values related to sociability, the value of work quality was also highlighted, considered a duty in the code of ethics and with direct impacts on the professionals’ attitude, the care provided, and the organization of routines.

Furthermore, perceptions of low social valuation of the profession and difficulties in reconciling individual values and institutional demands were identified. Issues such as overload and lack of autonomy also emerged in the focus groups as elements that hinder patient-centered care. These findings reinforce the importance of training strategies that encourage self-awareness and Values-Based Leadership, contributing to the strengthening of professional identity and the improvement of nursing management.

It is recommended that studies be conducted in different institutional contexts in order to deepen the understanding of the relationship between personal values, leadership styles, and organizational culture, as well as to assess the impact of educational interventions on leadership performance in nursing.

## Data Availability

The research data are available only upon request.
