# Supplementary material for: Perceptions of Nurses at a University Hospital regarding Personal Values and Leadership
Source: Rev Bras Enferm. 2026 Oct 26;79:e20250102. doi: 10.1590/0034-7167-2025-0102 (PMC13719133; doi:10.1590/0034-7167-2025-0102)
Supplement: Translation

# Translation: Percepções de Enfermeiros de Hospital Universitário sobre Valores Pessoais e Liderança

**DOI:** 10.1590/0034-7167-2025-0102pt

**Authors:** Chennyfer Dobbins Abi Rached, Giovanna Micucci Pires Amaral, Maria Luiza Prado Uelze, Bruna Franco Massa

**Affiliations:** I Universidade de São Paulo. São Paulo, São Paulo, Brasil

**Keywords:** Enfermagem, Enfermeiras e Enfermeiros, Liderança, Valores Sociais, Cuidados de Saúde Baseados em Valores.

## Abstract

**Objetivos:**

compreender as percepções dos enfermeiros gestores quanto aos valores pessoais e estilo de liderança.

**Métodos:**

pesquisa de campo qualitativa. Participaram 19 enfermeiros com cargo de gestão de hospital universitário em São Paulo. Realizaram-se cinco grupos focais com perguntas disparadoras que abordaram os estilos de liderança, os valores pessoais e o contexto institucional. Os dados foram analisados pelo método do Discurso do Sujeito Coletivo.

**Resultados:**

evidenciaram-se a prevalência do estilo relacional, a influência dos valores pessoais no estilo de liderança e conflitos entre valores pessoais e condições de trabalho.

**Considerações Finais:**

os valores pessoais se refletem na liderança, sendo o estilo relacional associado ao engajamento da equipe e à humanização no cuidado.

## INTRODUÇÃO

O conceito de liderança mundialmente disseminado é a capacidade de influenciar os outros em busca de objetivos comuns. John Maxwell, um dos autores mais influentes sobre liderança, afirma que “*The true measure of leadership is influence-nothing more, nothing less*”^(1)^. Diversas teorias e modelos de liderança definem o conceito da mesma forma^(2-5)^. Autores e pesquisadores na área de liderança e gestão sustentam que os valores pessoais dos líderes moldam a cultura da organização e influenciam o estilo de liderança^(6-9)^.

O conjunto de crenças e valores pessoais dos líderes explica as suas decisões e comportamentos, ou seja, funciona como um guia interno. Entre as maneiras pelas quais as crenças e os valores pessoais influenciam os líderes, podem-se destacar a tomada de decisão e um líder que valoriza a integridade prioriza decisões éticas. Durante situações de crise, os valores de um líder determinam o curso de ação. Líderes que valorizam a transparência se comunicam abertamente, enquanto aqueles que priorizam a segurança adotam posturas cautelosas^(9,10)^.

Líderes que valorizam a autodireção e benevolência adotam um estilo de liderança mais participativo e democrático, e constroem relacionamentos fortes e de apoio com suas equipes, promovendo um ambiente de trabalho positivo e colaborativo. Em contrapartida, aqueles que valorizam o poder costumam adotar um estilo mais autoritário e diretivo^(6-8)^.

Os valores pessoais oferecem *insights* sobre o comportamento dos líderes e, por sua vez, o impacto na prática profissional^(10,11)^. No contexto da enfermagem, líderes que valorizam a empatia são mais propensos a praticar a escuta ativa, essencial na interação com pacientes, famílias e equipes de saúde^(9,12)^.

A reflexão sobre o efeito dos valores pessoais no estilo de liderança possibilita o reconhecimento de preconceitos ou preferências pessoais que podem gerar conflitos ou tensões. Os valores orientam as interações, o que favorece o diálogo e trabalho colaborativo, contribuindo para maior engajamento dos trabalhadores, com efeitos positivos na assistência^(10,13)^.

Embora o fenômeno da liderança tenha sido vastamente estudado na área do comportamento organizacional, há uma lacuna no que se refere à relação entre a liderança e os valores pessoais e organizacionais. Diante disso, o foco deste estudo é compreender as percepções de enfermeiros gestores de hospital público universitário quanto aos valores pessoais e aos estilos de liderança. A investigação se baseia na Teoria dos Valores Pessoais de Schwartz e na Liderança Baseada em Valores, com o objetivo de contribuir para o fortalecimento de práticas gestoras conscientes, éticas e alinhadas à identidade profissional da enfermagem. Dessa forma, apresenta-se revisão bibliográfica que visa conceituar a estrutura de valores pessoais e sua influência no comportamento do indivíduo, bem como o desenvolvimento da Liderança Baseada em Valores.

### Teoria dos Valores Pessoais de Schwartz

A Teoria dos Valores Pessoais estabelece valores como “(a) princípios ou crenças, (b) sobre comportamentos ou estados de existência, (c) que transcendem situações específicas, (d) que guiam a seleção ou avaliação de comportamentos ou eventos e (e) que são ordenados por sua importância”^(14)^.

Segundo Schwartz (1992), os valores são classificados em dez tipos motivacionais: 1) universalismo; 2) benevolência; 3) conformidade; 4) tradição; 5) segurança; 6) poder; 7) realização; 8) hedonismo; 9) estimulação; e 10) autodeterminação. Tais valores apresentam graus distintos de sobreposição, o que, consequentemente, influencia a forma como o indivíduo se comporta e reage diante dos contextos apresentados^(8,15)^.

Sendo assim, o comportamento do indivíduo é influenciado pelo seu conjunto de valores pessoais e, por isso, o arcabouço de crenças e valores do líder impacta sua conduta^(9)^. Diante disso, líderes atentos aos valores dos membros de sua equipe contribuem para maior satisfação e engajamento. Portanto, os valores institucionais devem estar em sintonia com os valores do líder e dos colaboradores^(7,16,17)^.

### Liderança Baseada em Valores

Pensadores como Aristóteles e Kant discutiram a importância dos princípios éticos e da virtude, influenciando a noção de liderança moral, que consiste na ideia que líderes devem agir de acordo com valores éticos e morais^(7)^.

A Liderança Baseada em Valores emergiu como uma abordagem significativa na gestão e no estudo da liderança a partir da década de 1990, devido ao anseio por autenticidade e ética nos ambientes de trabalho. Ela se concentra na importância de os líderes serem guiados por valores fundamentais, o que influenciou o pensamento sobre como líderes podem ser eficazes e inspiradores quando possuem valores positivos^(4,7)^.

James MacGregor Burns, em 1978, publicou seu trabalho seminal “*Leadership*”, que introduziu a distinção entre liderança transformacional e transacional. A liderança transformacional, que se baseia em valores e inspira seguidores a alcançar objetivos elevados, foi uma das primeiras formas de liderança que enfatizou a importância dos valores pessoais^(2)^.

Bernard Bass expandiu e refinou as ideias de Burns, especialmente no contexto da liderança transformacional. A ênfase na visão e nos valores como fatores que motivam e inspiram os seguidores a alcançar desempenho elevado solidificou a base teórica da Liderança Baseada em Valores. Líderes do setor da saúde passaram a adotar esse modelo por meio de uma visão compartilhada e de valores fundamentais em prol do cuidado centrado no paciente^(2)^.

Nos anos 2000, a liderança autêntica ganhou destaque, com pesquisadores como Bill George, autor de “*Authentic Leadership*” (2003), argumentando que líderes eficazes são aqueles que agem com autenticidade e coerência em relação aos seus valores e princípios pessoais. Líderes autênticos são transparentes e constroem relações com empatia, respeito e confiança mútua^(13,17)^.

A liderança servidora, cujo autor é Robert K. Greenleaf, no ensaio “*The Servant as Leader*” (1970), influenciou a Liderança Baseada em Valores ao propor que uma liderança eficiente reúna valores como empatia e respeito, demonstrando comprometimento com o bem-estar dos seus seguidores^(3)^.

Sendo assim, a Liderança Baseada em Valores surge como uma resposta às demandas por maior ética, autenticidade e eficácia nos líderes. Os princípios éticos e morais, enquanto práticas de liderança, reforçam a importância de os líderes serem guiados por valores centrais que moldam suas ações. Essa abordagem tem sido influenciada por teorias de liderança transformacional, autêntica e servidora, bem como por mudanças culturais e contextos organizacionais, com destaque para a integridade e responsabilidade^(16,17)^.

Além disso, mudanças nas expectativas sociais e culturais em relação à responsabilidade social corporativa e à sustentabilidade também contribuíram para a popularização da Liderança Baseada em Valores, à medida que as organizações se tornaram mais conscientes de seu impacto socioambiental^(4,7,10)^. A Teoria da Liderança Baseada em Valores destaca que líderes eficazes agem de acordo com valores e princípios, promovendo confiança e engajamento entre os seguidores^(7,16,17)^.

Na área da saúde, a Liderança Baseada em Valores emergiu como resposta à busca por melhores práticas de gestão e por um cuidado ético e humanizado. A complexidade e o impacto direto das decisões de liderança sobre a saúde e o bem-estar das pessoas tornaram essencial a adoção de uma abordagem que priorize valores sólidos e princípios éticos^(2,9)^.

Diante de casos de má prática, negligência e falta de transparência, o anseio dos líderes por valores como segurança e qualidade no cuidado se tornou mais evidente. A Liderança Baseada em Valores tem sido aplicada com foco no cuidado centrado no paciente. Tais modelos enfatizam a importância de tratar os pacientes com dignidade e respeito, promovendo a participação ativa dos pacientes em suas próprias decisões de saúde. Nesse sentido, os valores dos enfermeiros influenciam a assistência recebida pelos pacientes e suas famílias^(2,9)^.

## OBJETIVOS

Compreender as percepções de enfermeiros gestores quanto aos valores pessoais e estilos de liderança.

## MÉTODOS

### Aspectos éticos

Este estudo foi aprovado pelo Comitê de Ética em Pesquisa da Escola de Enfermagem da Universidade de São Paulo (Certificado de Apresentação para Apreciação Ética (CAAE) n^o^ 51073421.2.1001.5392) e do Hospital Universitário (CAAE n^o^ 51073421.2.3001.0076). Todos os participantes assinaram o Termo de Consentimento Livre e Esclarecido, conforme preconizado pela Resolução n^o^ 466/12 do Conselho Nacional de Saúde.

### Tipo de estudo

Trata-se de estudo qualitativo de natureza exploratória, fundamentado nos critérios do *COnsolidated criteria for REporting Qualitative research*, com enfoque na compreensão das percepções dos enfermeiros gestores quanto aos valores pessoais e ao estilo de liderança adotado. A coleta e análise dos dados seguiram o método do Discurso do Sujeito Coletivo (DSC)^(18)^, permitindo a representação das ideias centrais coletivas a partir das falas individuais.

### Procedimentos metodológicos

Os participantes foram convidados por critério de amostragem intencional, através de e-mail vinculado ao serviço de educação permanente. Dessa forma, dois enfermeiros recusaram o convite, participando deste estudo 19 enfermeiros com cargos formais de liderança (coordenador, chefe ou diretor de departamento assistencial) de hospital universitário localizado em São Paulo, Brasil, com 258 leitos de capacidade instalada. O critério de inclusão foi ocupar cargo de liderança na assistência de enfermagem, com experiência mínima de um ano na função. É válido ressaltar que, durante a pesquisa, não houve desistência por parte dos participantes e que estes não possuíam relacionamento prévio com a equipe pesquisadora.

### Instrumentos e coleta de dados

A coleta ocorreu em duas etapas. Inicialmente, foram aplicados dois instrumentos: (1) a Escala de Avaliação do Estilo Gerencial (EAEG)^(19)^, composta por 19 itens em escala Likert (1 a 5), que identifica os estilos de liderança predominantes (relacional, tarefa ou situacional); e (2) o *Culture and Value Analysis Tool* (C-VAT)^(20)^, uma escala ipsativa baseada na Teoria dos Valores Pessoais de Schwartz, que classifica os valores em quatro dimensões (trabalho, relações, controle e pensamento), com subdimensões e pontuação de 5 a 20 pontos.

Posteriormente, foram realizados cinco grupos focais, com duração média de 90 minutos cada, conduzidos semanalmente em espaço reservado da instituição. A mediação foi conduzida por pesquisadoras experientes com atuação na docência e observadoras não participantes, todas da área da enfermagem. Destaca-se que a equipe pesquisadora recebeu treinamento da coordenadora da pesquisa. Durante os grupos, foram feitas notas de campo e também realizadas gravações em áudio. A transcrição literal do conteúdo dos grupos focais foi realizada.

As questões disparadoras foram “Quais estilos de liderança vocês identificam como mais frequentes na enfermagem?”, “Quais valores pessoais influenciam sua forma de liderar?”, “Vocês percebem relação entre seus valores pessoais e o estilo de liderança adotado?”, “Como esses valores impactam o relacionamento com a equipe?” e “De que forma o ambiente institucional favorece ou limita a expressão desses valores?”. Os encontros também contemplaram momentos de reflexão com base nos resultados individuais das escalas aplicadas. Não houve repetições das entrevistas, e a saturação de dados foi discutida entre os membros da equipe pesquisadora.

### Análise dos dados

As transcrições dos grupos focais foram analisadas segundo o método do DSC, conforme proposto por Lefèvre e Lefèvre^(18)^, que permite identificar expressões-chave, ideias centrais e elaborar discursos-síntese representativos da coletividade. Essa abordagem foi triangulada com os resultados obtidos nas escalas EAEG e C-VAT, o que possibilitou uma interpretação mais aprofundada das percepções e dos valores manifestados pelos participantes. A triangulação dos dados foi empregada para garantir maior rigor e validade interpretativa. Ressalta-se que os temas emergidos ao longo da análise foram derivados dos dados.

### Rigor metodológico

Para assegurar a fidedignidade da análise, foram utilizados os critérios de credibilidade, transferência, dependência e confirmação, conforme o referencial de Lincoln e Guba^(21)^. Todas as etapas foram registradas em caderno de campo, com duplo código dos dados e revisão independente das transcrições. As transcrições não foram entregues aos participantes, mas a devolutiva dos discursos aos participantes, para validação das interpretações (*member checking*), foi realizada ao final dos encontros, possibilitando reflexões a partir dos achados e também o *feedback* dos participantes para a equipe pesquisadora.

## RESULTADOS

A análise dos grupos focais revelou três eixos principais relacionados à percepção dos enfermeiros sobre estilos de liderança e valores pessoais: (1) prevalência do estilo relacional; (2) influência dos valores pessoais na prática de liderança; e (3) tensões entre valores pessoais e contexto institucional.

Quanto ao primeiro eixo, a maioria dos participantes apontou o estilo de liderança baseado em relações como o mais evidente e desejável na prática da enfermagem, por promover um ambiente de trabalho acolhedor, empático e colaborativo. As falas destacaram a empatia, a escuta, o respeito às diferenças e o cuidado com o outro como fundamentos dessa liderança. *“Cooperação e respeito transformam as equipes, motivando a colaboração entre todos”*, relatou o Sujeito 12, demonstrando a compreensão do valor relacional como estruturante do cuidado.

Em contrapartida, um grupo menor valorizou o estilo gerencial focado em tarefas, relacionado à necessidade de controle, eficiência e padronização dos processos. Participantes como o Sujeito 5 argumentaram que esse modelo assegura o cumprimento de prazos e protocolos, o que impacta diretamente a segurança do paciente. Embora menos frequente, esse estilo foi compreendido como complementar à liderança relacional, sobretudo em contextos de alta demanda.

O estilo situacional também foi reconhecido, especialmente entre os gestores com altos índices de flexibilidade na escala C-VAT. Os discursos enfatizaram a necessidade de adaptar o comportamento do líder à realidade da equipe e ao contexto assistencial, evidenciado por falas como a do Sujeito 17: *“O líder precisa ser flexível e tomar as decisões conforme o momento, a situação e o perfil da equipe”.*

O segundo eixo analítico demonstrou a forte influência dos valores pessoais sobre a prática de liderança. A dimensão “trabalho” obteve as maiores pontuações no C-VAT, com ênfase na subdimensão “qualidade”, destacada como pilar ético e técnico da liderança em enfermagem. O valor “qualidade” foi associado tanto ao cuidado com o paciente quanto à organização das rotinas de trabalho.

A dimensão “relações” também foi fortemente valorizada, especialmente a subdimensão “sociabilidade”. Os participantes destacaram que a comunicação efetiva, a empatia e o acolhimento promovem um clima organizacional positivo, favorecendo o engajamento da equipe. *“A sociabilidade facilita a troca de conhecimento e melhores práticas com a equipe multiprofissional”,* afirmou o Sujeito 21.

Em contrapartida, a subdimensão “*status*” obteve as pontuações mais baixas, evidenciando a percepção de desvalorização da profissão. Falas como a do Sujeito 3 (*“A sociedade não valoriza o enfermeiro, e nós mesmos não nos valorizamos”*) indicam a internalização desse estigma profissional, o que pode impactar negativamente a identidade e autoimagem dos enfermeiros.

Por fim, o terceiro eixo indicou uma tensão entre os valores pessoais dos enfermeiros-líderes e as condições institucionais que limitam sua expressão. Ainda que os participantes reconheçam a importância da escuta, do planejamento e do acolhimento, mencionaram que a sobrecarga de trabalho, a falta de autonomia e a cultura organizacional hierarquizada dificultam a vivência plena desses valores no cotidiano.

Esses resultados apontam para a necessidade de programas institucionais que promovam o alinhamento entre os valores dos profissionais e a cultura organizacional, criando espaços para o exercício de uma liderança coerente com os princípios da enfermagem e com a humanização do cuidado.

## DISCUSSÃO

Os achados deste estudo indicam que a liderança focada nas relações é predominante entre os enfermeiros gestores do hospital universitário investigado. As falas dos participantes destacaram que valores como empatia, afeto, respeito e confiança moldam a forma como esses profissionais lideram suas equipes. Tais percepções se alinham à literatura sobre liderança transformacional, que enfatiza o empoderamento, a autonomia e o reconhecimento das contribuições individuais como elementos centrais de uma gestão eficaz^(2,22)^. Por exemplo, o discurso do Sujeito 20, que destaca a importância do afeto e do respeito, reflete diretamente a essência dessa abordagem.

Embora o estilo relacional tenha sido majoritariamente defendido, alguns gestores apontaram a liderança com foco em tarefas como a mais apropriada em contextos que exigem o cumprimento rigoroso de prazos e metas. As falas do Sujeito 1 e Sujeito 5 ilustram essa perspectiva, ao enfatizarem a relevância da padronização e da segurança no cuidado. Esse perfil se aproxima da liderança transacional, caracterizada pela orientação para resultados, uso de instruções claras e foco no desempenho^(23,24)^.

A liderança situacional também foi mencionada entre os participantes, sobretudo entre aqueles que demonstraram altos índices de flexibilidade na escala C-VAT. Os discursos do Sujeito 8 e do Sujeito 17 evidenciaram essa adaptabilidade, reafirmando a teoria de Hersey e Blanchard^(13)^, segundo a qual a eficácia do líder reside na capacidade de ajustar o estilo às necessidades da equipe e às condições do ambiente.

Ao analisar os resultados da escala C-VAT, observou-se destaque para as dimensões “trabalho” e “relações”. O valor “qualidade”, fortemente citado nas falas, aparece como norteador das práticas dos enfermeiros-líderes, sendo também um dever ético profissional conforme o Código de Ética da Enfermagem^(25)^. O Sujeito 9, por exemplo, associou diretamente a qualidade ao bom relacionamento com os pacientes e à eficácia do tratamento.

Na dimensão “relações”, a subdimensão “sociabilidade” foi amplamente valorizada. As falas dos participantes reforçaram que a sociabilidade favorece a comunicação, o acolhimento e a troca de experiências no ambiente multiprofissional. Isso dialoga com estudos que apontam a empatia como elemento-chave na liderança em enfermagem, na comunicação com o paciente e suas famílias e na humanização do cuidado^(12,26-28)^. Considerando que o líder influencia o modo como as relações se estabelecem, vale acentuar que tais aspectos relacionais também impactam a segurança do paciente^(29,30)^.

Por outro lado, a subdimensão “*status*” recebeu as menores pontuações, refletindo uma percepção de baixa valorização social da profissão. As falas do Sujeito 3 e Sujeito 15 expressaram uma sensação de invisibilidade e luta por reconhecimento, corroborando estudos que denunciam os estereótipos e a desvalorização da enfermagem na mídia e na sociedade^(31-33)^.

A subdimensão “planejamento”, por sua vez, foi bem avaliada, sendo considerada essencial para a organização da assistência e para a segurança do paciente. Os dados sugerem que o enfermeiro-líder valoriza o planejamento como parte integrante do Processo de Enfermagem, aspecto também respaldado pelas normativas do Conselho Federal de Enfermagem^(34)^. Nesse sentido, o enfermeiro-líder influencia não só a assistência prestada, mas também a forma como ela é recebida pelos pacientes e suas famílias, tendo em vista que o cuidado mais seguro e fundamentado resulta em maior reconhecimento da equipe de enfermagem^(35)^.

### Limitações do estudo

Este estudo apresenta limitações inerentes ao delineamento qualitativo e ao contexto investigado. A amostra foi intencional, composta por 19 enfermeiros com cargos de gestão de um único hospital universitário, o que restringe a transferibilidade dos achados para outros serviços, regiões e modelos de governança. A coleta em grupos focais pode ter sido influenciada por dinâmicas grupais, hierarquia percebida e desejabilidade social, favorecendo consensos e inibindo posicionamentos divergentes, apesar das estratégias adotadas para rigor. Trata-se de um recorte transversal, que capta percepções em um momento específico e não permite inferir mudanças ao longo do tempo nem relações causais entre valores pessoais e estilos de liderança.

### Contribuições para a área da enfermagem, saúde ou políticas públicas

Refletir acerca dos valores pessoais e de como estes influenciam os comportamentos dos enfermeiros-líderes possibilita análises das experiências pessoais e dos pensamentos, além de permitir o repensar dos valores pessoais e profissionais.

Os encontros desencadearam um processo de autoconhecimento nos enfermeiros quanto aos seus valores, visto que repensá-los é um processo reflexivo que demanda tempo. Os valores impactam o estilo de liderança adotado e, consequentemente, a cultura e o clima organizacional. Conhecer os próprios valores e ter a oportunidade de repensá-los favorece transformações.

Para a organização, compreender o perfil de valores pessoais e o estilo de liderança de seus trabalhadores pode representar um ponto de partida para refletir sobre a direção a ser seguida, contribuindo para o planejamento de programas de gestão de cultura e clima organizacional, bem como para o desenvolvimento da Liderança Baseada em Valores com foco nos resultados identificados.

## CONSIDERAÇÕES FINAIS

Os achados deste estudo evidenciam que os valores pessoais influenciam o estilo de liderança adotado. Os valores mais destacados - empatia e sociabilidade - estão diretamente associados ao estilo de liderança relacional, que se mostrou predominante entre os enfermeiros. Esses valores demonstram potencial para fortalecer o clima organizacional e qualificar a assistência prestada, especialmente no que diz respeito à humanização do cuidado.

Além dos valores relacionados à sociabilidade, destacou-se também o valor da qualidade do trabalho, considerado um dever no código de ética e com impactos diretos na postura dos profissionais, no cuidado prestado e na organização das rotinas.

Ademais, foram identificadas percepções de baixa valorização social da profissão e dificuldades na conciliação entre valores individuais e exigências institucionais. Questões como sobrecarga e falta de autonomia também emergiram nos grupos focais como elementos que dificultam uma assistência centrada no paciente. Esses achados reforçam a importância de estratégias formativas que estimulem o autoconhecimento e a Liderança Baseada em Valores, contribuindo para o fortalecimento da identidade profissional e para o aprimoramento da gestão em enfermagem.

Recomenda-se a realização de estudos em diferentes contextos institucionais, com o intuito de aprofundar a compreensão sobre a relação entre valores pessoais, estilos de liderança e cultura organizacional, bem como avaliar o impacto de intervenções educativas sobre o desempenho das lideranças na enfermagem.

## Data Availability

Os dados de pesquisa só estão disponíveis mediante solicitação.
